# 

**Published:** 1887-07

**Authors:** 


### CONTENTS.

ORIGINAL COMMUNICATIONS—

Alcoholic Liquors in the Practice of Medicine. By Eugene Foster, M. D., Augusta, Ga. . . . . 257
Cholera Infantum. By James B. Baird, M. D., Atlanta, Ga. . . . . 271
Alcohol Poisoning. By W. B. Parks, M. D., Atlanta, Ga. . . . . 282

CLINIC REPORTS—

A Case in Practice Supposed to be an Abscess of the Brain. By Howard J. Williams, A. M. M. D., Macon, Ga. . . . . 286

SOCIETY REPORTS—

Chicago Medical Society . . . . . 290

CORRESPONDENCE.

Our New York Letter . . . . . 304
Dr. Gaston Explains . . . . . 307
Letter from a Country Doctor . . . . . 309

BOOK REVIEWS—

Practical Treatise on Obstetrics. By A. Carpentier, M. D., Paris, 311

EDITORIAL—

Criminal Abortion . . . . . 314
The Cost of Caring for the Sick Poor of Atlanta . . . . . 316
Alcoholic Liquors in the Practice of Medicine . . . . . 320
Items . . . . . 285, 289, 303, 320
Our Advertisers . . . . . 323

